# Continuous Research of Headache and Migraine

**DOI:** 10.3390/neurolint16040059

**Published:** 2024-07-22

**Authors:** Yasushi Shibata

**Affiliations:** Department of Neurosurgery, Mito Medical Center, University of Tsukuba, Mito Kyodo General Hospital, Miyamachi 3-2-1, Ibaraki 310-0015, Japan; yshibata@md.tsukuba.ac.jp

## 1. Introduction

Headache is a common disorder with high prevalence. Migraine is known to heavily disturb the daily life of patients. So, the direct social and indirect economic losses caused by absenteeism and presenteeism have been reported to be huge. Recent developments in new drug therapies are specific and effective for most patients with migraine. However, there are still some patients whose condition cannot be controlled using current therapies. We need continuous research to effectively use these new medications. 

## 2. Discussion

This Special Issue includes a variety of basic, translational, and clinical research and case reports. A step-by-step understanding of migraine pathophysiology is crucial for clinical decision making and future research. 

Because there are no clinically available diagnostic biomarkers of migraine, the diagnosis and evaluation of patients’ headaches has solely depended on interviews regarding their clinical history and symptoms. Migraine biomarkers are a hot area of research. Some neuropeptides could be commercially available markers of migraine. Another biomarker may be imaging methods such as magnetic resonance imaging (MRI). Studies of these biomarkers could provide effective diagnostic tools and indicators of the effects of treatment.

We understand that genetics is also involved in the various forms of headache including migraine. Some specific genetic diseases are accompanied by clinical migraine. However, a single gene causative of migraine has not been demonstrated. Genetic studies of headache disorders will identify the causal mechanisms of those genes and clinical headache. These studies will provide fundamental therapy strategies for these headaches.

Clinical studies and clinical case reports provide insight for daily clinical practice. We can use many drugs and some interventions, such as neuro-stimulation. These therapies have been effective in some patients. In clinical practice, we must select these therapies according to each patient. These therapeutic strategies, including their ideal combinations, need more research.

## 3. Conclusions

I hope this Special Issue contributes to the current understanding of migraine and headache. Readers may acquire inspiration for future studies from the research articles in this Special Issue. 

